# Developmental Differences in Probabilistic Reversal Learning: A Computational Modeling Approach

**DOI:** 10.3389/fnins.2020.536596

**Published:** 2021-01-18

**Authors:** Eileen Oberwelland Weiss, Jana A. Kruppa, Gereon R. Fink, Beate Herpertz-Dahlmann, Kerstin Konrad, Martin Schulte-Rüther

**Affiliations:** ^1^Translational Brain Research in Psychiatry and Neurology, Department of Child and Adolescent Psychiatry, Psychosomatics, and Psychotherapy, University Hospital Aachen, Aachen, Germany; ^2^Cognitive Neuroscience, Institute of Neuroscience and Medicine (INM-3), Jülich Research Centre, Jülich, Germany; ^3^Institute of Neuroscience and Medicine (INM-11), Jülich Research Centre, Jülich, Germany; ^4^Child Neuropsychology Section, Department of Child and Adolescent Psychiatry, Psychosomatics, and Psychotherapy, University Hospital Aachen, Aachen, Germany; ^5^Department of Neurology, University Hospital Cologne, Cologne, Germany; ^6^Department of Child and Adolescent Psychiatry, Psychosomatics, and Psychotherapy, University Hospital Aachen, Aachen, Germany; ^7^Department of Child and Adolescent Psychiatry and Psychotherapy, University Medical Center Göttingen, Göttingen, Germany

**Keywords:** cognitive flexibility, executive functioning, development, reinforcement learning, feedback processing

## Abstract

Cognitive flexibility helps us to navigate through our ever-changing environment and has often been examined by reversal learning paradigms. Performance in reversal learning can be modeled using computational modeling which allows for the specification of biologically plausible models to infer psychological mechanisms. Although such models are increasingly used in cognitive neuroscience, developmental approaches are still scarce. Additionally, though most reversal learning paradigms have a comparable design regarding timing and feedback contingencies, the type of feedback differs substantially between studies. The present study used hierarchical Gaussian filter modeling to investigate cognitive flexibility in reversal learning in children and adolescents and the effect of various feedback types. The results demonstrate that children make more overall errors and regressive errors (when a previously learned response rule is chosen instead of the new correct response after the initial shift to the new correct target), but less perseverative errors (when a previously learned response set continues to be used despite a reversal) adolescents. Analyses of the extracted model parameters of the winning model revealed that children seem to use new and conflicting information less readily than adolescents to update their stimulus-reward associations. Furthermore, more subclinical rigidity in everyday life (parent-ratings) is related to less explorative choice behavior during the probabilistic reversal learning task. Taken together, this study provides first-time data on the development of the underlying processes of cognitive flexibility using computational modeling.

## Introduction

In an ever-changing environment, it is essential to shift strategies and adapt response patterns based on received feedback. Probabilistic reversal learning tasks have been effectively used to assess cognitive flexibility, since they require participants to learn rules in an uncertain environment while remaining flexible in response to changing rules, a capacity particularly relevant to socio-emotional behavior (Cools et al., 2002; Remijnse et al., 2005; Ghahremani et al., 2010; Nashiro et al., 2012; Hauser et al., 2015b; Izquierdo et al., 2016). In a probabilistic reversal learning task, participants learn to identify the target amongst various simultaneously presented stimuli based on received feedback. After participants have successfully learned to identify the target, a reversal will occur, and the previously non-rewarded stimulus will become the new target. Importantly, the stimulus-feedback contingencies are not fixed and deterministic, but probabilistic, i.e., a stimulus receives a certain rewarding feedback not with each presentation or choice, but only with a certain probability. In addition, reversal learning has been found to be impaired in various neurological and psychiatric conditions including obsessive compulsive disorder (OCD; Verfaillie et al., 2016; Hauser et al., 2017; Tezcan et al., 2017), Huntington’s Disease (Nickchen et al., 2016), schizophrenia (Culbreth et al., 2016; Reddy et al., 2016), Parkinson’s disease (Buelow et al., 2015), Attention-Deficit/Hyperactivity disorder (ADHD; Hauser et al., 2015a), and autism spectrum disorder (ASD; Lionello-DeNolf et al., 2010; D’Cruz et al., 2013; Costescu et al., 2014; D’Cruz et al., 2016). Despite these numerous studies on cognitive flexibility, it remains difficult to draw exact conclusions about the development of the underlying learning processes mainly due to three crucial factors: (1) In the existing studies participants varied in age from young childhood to adulthood with only one study systematically comparing learning processes at various ages during development (Crawley et al., 2019), although it is known that cognitive flexibility changes over the course of development (e.g., Crone and van der Molen, 2004; Yurgelun-Todd, 2007; Van Der Schaaf et al., 2011; Ionescu, 2012; Luking et al., 2014). (2) Most current studies made their conclusions based on error scores as an observable index of reversal learning. However, these do not provide nuanced information needed to infer how the underlying mechanisms of cognitive flexibility (as measured by reversal learning) are implemented. (3) The feedback used in the existing studies differed from winning pieces of candy to points and money with not a single study comparing the effect of these different types of feedback on reversal learning. Yet, we know that behavior heavily depends on the received feedback and that feedback processing also changes during the course of development (e.g., Eppinger and Kray, 2009; van den Bos et al., 2012; Van Duijvenvoorde et al., 2013). We will shortly outline these three aspects before proposing how to overcome these limitations in the current study.

First, existing developmental studies on cognitive flexibility, including behavioral and neuroscientific studies, typically only included either children or adolescents, but by comparing between studies developmental differences between age-groups can be found. That is, younger children usually make many errors and show preservative behavior (Landry and Al-Taie, 2016), while adolescents often show more riskiness resulting in poor decisions (Van Der Schaaf et al., 2011; Blakemore and Robbins, 2012; Hauser et al., 2015b), possibly due to a hypersensitive system for processing rewards (Somerville et al., 2010). However, these studies did not use probabilistic reversal learning tasks, which may better capture the essence of cognitive flexibility because they provide more ecological validity with respect to unstable learning environments. Furthermore, due the diverse nature of the employed tasks across developmental ages, conclusions with respect to developmental effects remain speculative. Hence, directly comparing children and adolescents using a probabilistic reversal learning task is essential to bring about a clearer understanding of the development of cognitive flexibility.

Second, probabilistic reversal learning tasks inherently bring about relatively high numbers of errors. Therefore, probabilistic reversal learning tasks need a more nuanced measurement of how participants integrate information throughout the task than simple observable error rates. Computational models of behavior specify parameters that are independent of task structure (as in the case of probabilistic reversal learning task the trial-and-error structure typically results in high number of errors), and are therefore better suited to approximate the underlying mechanisms governing the observed behavior (i.e., error patterns). Computational modeling could bridge the gap between observable behavior and internal mechanisms by using biologically plausible models to infer the psychological mechanisms underlying typical and deficient learning processes (e.g., Weeda et al., 2014; Palminteri et al., 2016; Schuch and Konrad, 2017).

A relatively simple approach is to use reinforcement learning models which assume that future choices are optimized via maximization of favorable outcomes being acquired using a fixed learning rate and reward prediction errors (Rescorla and Wagner, 1972). However, such simplistic models do not consider learning under uncertainty. During probabilistic reversal learning though, individuals have to learn in a highly unstable environment, implicating that the learning rate adjusts according to its estimates of environmental volatility. More recently, hierarchical Gaussian filter models (HGF), which are hierarchical Bayesian learning models, have been used to model individual learning under multiple forms of uncertainty (Mathys et al., 2011), since they can dynamically adjust their learning rate according to their estimates of environmental volatility (i.e., the probability contingencies may change at any point), its uncertainty about the current state, and/or any perceptual uncertainty (Jiang et al., 2014). Thus, they also provide more temporally rich information about the dynamics of learning by considering trial-by-trial information instead of averaged error rates and reaction times. For instance, Hauser et al. (2014) could demonstrate that adolescents with ADHD performed marginally worse than their typically developing (TD) peers in standard measures of behavior (i.e., error rates, RTs) while computational modeling revealed that although both groups had similar learning rates, the ADHD group had more “explorative” tendencies in their choice-behavior resulting in less efficient task performance. In sum, computational models might thus provide the more nuanced measurement that is needed for probabilistic reversal learning tasks to understand the underlying mechanisms and its development.

Third, even though most (probabilistic) reversal learning tasks have a comparable design with respect to timing and feedback contingencies, the type of feedback differs substantially between, but not within studies (Cools et al., 2002; Remijnse et al., 2005; Ghahremani et al., 2010; Nashiro et al., 2012; D’Cruz et al., 2013; Hauser et al., 2014; Buelow et al., 2015). Since previous studies on feedback processing have revealed different effects, drawing an unambiguous conclusion here is yet impossible. While one study demonstrated that children show better results in a go/no-go task with monetary compared to social reward (Kohls et al., 2009), others have not found this effect, but investigated children and adolescents (Demurie et al., 2011). More homogenously, previous studies in clinical samples (i.e., participants with ASD and ADHD) demonstrated that non-social reward (e.g., arrows, money) appears to be more effective than social reward (Demurie et al., 2011; Stavropoulos and Carver, 2014). Hence, directly examining the effect of different types of feedback in children *and* adolescents may bring a clearer understanding of the development of feedback processing in the domain of cognitive flexibility.

The main aim of this study was to investigate developmental differences in learning processes underlying cognitive flexibility and the effect of various types of feedback using a probabilistic reversal learning task in children and adolescents. The secondary aim was to explore possible relationships with subclinical measures of ASD symptomatology, with a specific interest in restrictive and repetitive behavior, since it has been proposed that impairments in cognitive flexibility may contribute to this domain (South et al., 2005; D’Cruz et al., 2013). We therefore (1) tested a sample of children and adolescents in order to compare both age groups; (2) used various kinds of feedback including social (i.e., an actor posing thumbs up and smiling versus an actor gazing straight with a neutral expression), individual (i.e., favorite hobby of each participant versus pixelated video of the hobby), and control (i.e., a check mark versus a cross) feedback within participants performing a probabilistic reversal learning task; and (3) used reinforcement learning and HGF models to infer the psychological mechanisms underlying the learning processes and potential individual differences. Finally, we also included measures of subclinical ASD symptomatology.

## Materials and Methods

### Participants

In total, 28 TD children (all male, 8–12 years of age, mean age = 10.33 years) and 25 TD adolescents (all male, 13 to 17 years of age, mean age = 15.57 years) were included in the final analyses. An additional two children participated in the study, but were not included in the analyses because they were not able to complete all experimental runs due to fatigue/non-compliance.

All participants had no indication of developmental delay or other psychiatric disorders as assessed by a structured screening interview on the phone and the Child Behavior Checklist (CBCL, Achenbach, 1991; all *T* < 65). Only participants with sufficient cognitive abilities were included [IQ > 80, short version of the Wechsler Intelligence Scale for children or Grundintelligenztest Skala (CFT-20R; Weiß, 2006)]. The study was approved by the ethics committee of the university. All participants and/or their caregivers gave written informed consent/assent to participate in the study. Participation was compensated by money irrespective of task performance. All participants were seated in a quiet lab.

### Probabilistic Reversal Learning Task

**FIGURE 1:**
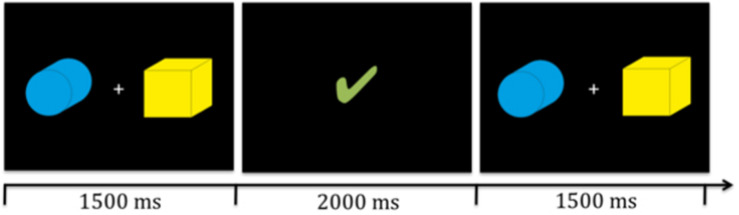
Illustration of two target stinmuli (boxes) and timing. Participants were presented with the stimuli for 1500 ms in which they had to give their response via a key press. Then the reward video (illustrated here by the green check mark) was presented for 2000 ms. Subsequently, the next trial began.

We designed a probabilistic reversal learning task in which participants had to learn to identify the target amongst two stimuli presented simultaneously on the screen (see Figure 1) based on feedback.

Participants received 75% contingent feedback (i.e., rewarding feedback upon correct choice and non-rewarding feedback upon incorrect choice) and 25% non-contingent feedback (i.e., non-rewarding feedback upon correct choice and rewarding feedback upon incorrect choice). After participants reached a learning criterion, the other stimulus, which was previously not rewarded, became the new rewarded target without giving notice to the participant (i.e., reversal). The learning criterion was reached when participants had completed at least six to ten trials (randomly assigned during each reversal block) and had identified the target correctly in three consecutive trials. Participants were familiarized with the task and stimuli before testing. They were informed that after a while a reversal of targets could occur, and that feedback was given probabilistically but they were given no explicit information about the learning criterion or the ratio of contingent and non-contingent feedback. During the practice session, feedback was given exactly as during the experiment but without any reversal.

All participants were seated approximately 30 cm in front of the computer and presented with identical stimuli (see Figure 1). The stimuli were of identical size (approximately 9.5 cm × 9.5 cm and 17° visual angle) and were presented simultaneously to the left and right side of a fixation cross on the screen. The position of the targets switched randomly with replacement, thus it was possible that the target appeared on the same position multiple times in row. All participants completed three runs of the probabilistic reversal learning task with varying feedback per run; i.e., social, individual, and control feedback, in a counterbalanced order. The feedback videos were presented visually for 2000 ms. Five different videos in each feedback condition were used. Each run included 12 reversals or a maximum of 240 trials. For a detailed overview of the timing of one trial see Figure 1. If participants did not react within 1500 ms, the fixation cross turned red and subsequently the next trial started. All missed trials were excluded from final analyses. The maximum numbers of total misses per condition did not exceed 17.

#### Social Feedback (SF) Video

**FIGURE 2:**
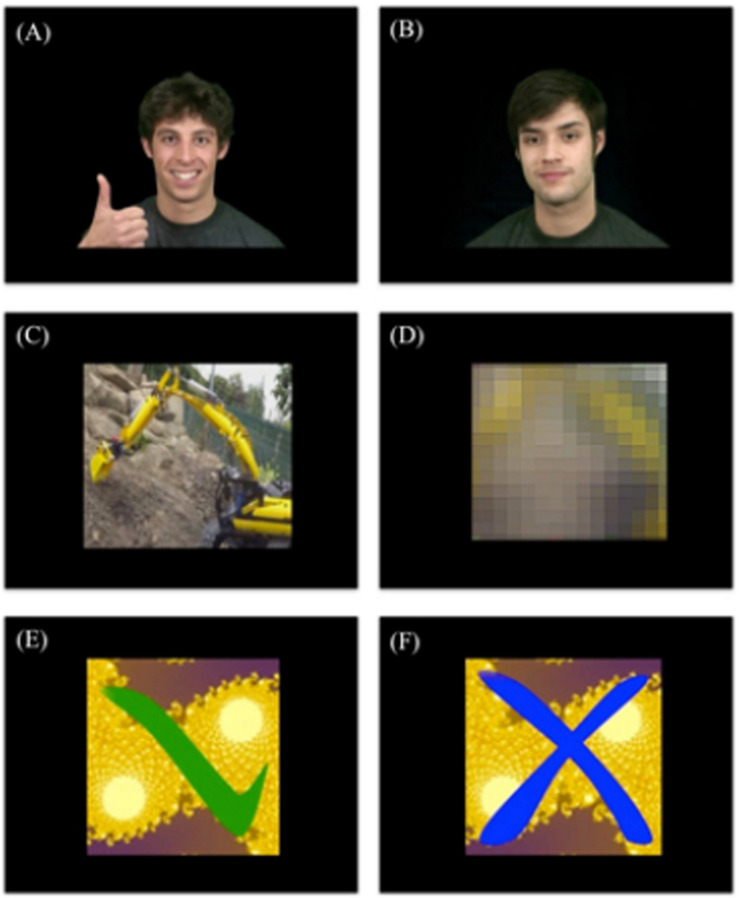
Examples of feedback videos. **(A)** Rewarding social feedback video, **(B)** non-rewarding social feedback video, **(C)** rewarding individualized feedback video, **(D)** non-rewarding individualized feedback video, **(E)** rewarding control feedback video, and **(F)** non-rewarding control feedback video.

Videos are part of a larger pool that has been designed for previous studies on social reward (Kohls et al., 2013; Chevallier et al., 2016). Five individuals were used, both for the rewarding and non-rewarding feedback. The rewarding feedback videos depicted individuals looking straight at the participants, smiling, and giving thumbs up (see Figure 2A) whereas the non-rewarding feedback videos depicted the individuals gazing straight at the participant with a neutral facial expression and no hand movement (see Figure 2B).

#### Individualized Feedback (IF) Video

Five video clips depicting a favorite activity of daily living of the participant were created (e.g., computer game, movie, series, sports club etc.; see Figure 2C) and used as the rewarding feedback videos. For the non-rewarding feedback, the rewarding feedback videos were manipulated so that they were completely unrecognizable to the participant (see Figure 2D), while keeping basic visual stimulation identical. We used Adobe Premiere Pro CS5.5 to add three effects to each video in order to make it unrecognizable: (1) Gaussian Blur (165), (2) Mosaic (horizontal 29 and vertical 25), and (3) Sharpen (764).

#### Control Feedback (CF) Video

The rewarding feedback videos depicted a green tick mark (see Figure 2E) and the non-rewarding feedback (see Figure 2F) videos depicted a blue cross, both appearing on colored fractal images. Five different backgrounds were selected so that the same number of different videos was presented for all feedback conditions.

The software Presentation 9 (Neurobehavioral Systems, Albany, CA, United States^1^) was used for stimulus presentation and response collection. After task completion all participants rated each video (in total 30 videos) on a 10-point Likert scale ranging from 0 (non-rewarding) to 10 (very rewarding).

### Questionnaires

The parent of each participant filled out the Social Responsiveness Questionnaire (SRS, Constantino et al., 2003). This questionnaire is designed to assess the severity of autism spectrum symptoms from subclinical characteristics to highly impaired social skills. We were specifically interested in the subscale “restrictive interests and repetitive behavior,” since it reflects rigid behavior and stereotypes typically observable in children with ASD, but also subclinical variance in TD children.

### Analyses

All behavioral data were analyzed using MATLAB 8.1 and IBM SPSS Statistics 21. First, Mann-Whitney tests were computed to compare the parameter estimates of the model. Second, General Linear Model (GLM; univariate and repeated-measures) analyses were computed in order to assess main effects and interactions of various error rates (within-group factors: Social/Individualized/Control Feedback; between-group factor: age-group). *Post hoc t*-tests were performed to determine differences between conditions. Additional ANOVAs with a variable specifying the order of each feedback (i.e., social feedback as first, second or third run, individual feedback as first, second or third run and control feedback as first, second or third run) as a factor were computed to check for order effects.

We made the data and code for the model and descriptive analyses publicly available: Open Science Foundation^2^.

#### Computational Models

We then computed a simplistic anti-correlated Rescorla-Wagner (RW) model, which has also been used to infer learning in probabilistic reversal learning tasks (Gläscher et al., 2009; Hauser et al., 2014) and two versions of a hierarchical Gaussian filter (HGF) model, which specifically considers learning in an uncertain environment as in the case of a probabilistic reversal learning task (Hauser et al., 2014). We compared all three models (see below) using Bayesian model comparison to quantify which of these models best explained the observable behavior (a) separately within both age groups and (b) across age groups.

##### Anti-correlated rescorla-wagner (RW) learning model

The RW model has a fixed learning rate across the whole experiment. The reward prediction error (RPE) δ at each trial (t) was computed as the difference between the anticipated (V^(t)^_*chosen*_) and the received (R^(t)^) outcome:

$$\delta^{\left(t\right)}=R^{\left(t\right)}-V_{c\,h\,o\,s\,e\,n}^{\left(t\right)}$$

Previous studies have suggested that individuals also use the counterfactual information where always one choice is correct, the other is incorrect, to update their stimulus-reward association. Therefore, we applied the anti-correlated RW model, an extension of the standard RW model, where the values of both options, chosen and unchosen (i.e., box 1 and box 2, see Figure 1) were updated using the RPE δ:

$$V_{c\,h\,o\,s\,e\,n}^{\left(t+1\right)}=V_{c\,h\,o\,s\,e\,n}^{\left(t\right)}+\alpha \,\delta^{\left(t\right)}$$

$$V_{u\,n\,c\,h\,o\,s\,e\,n}^{\left(t+1\right)}=V_{u\,n\,c\,h\,o\,s\,e\,n}^{\left(t\right)}-\alpha \,\delta^{\left(t\right)}$$

where α depicts the learning rate, which is constant throughout the experiment.

##### Hierarchical gaussian filter (HGF) models

**FIGURE 3:**
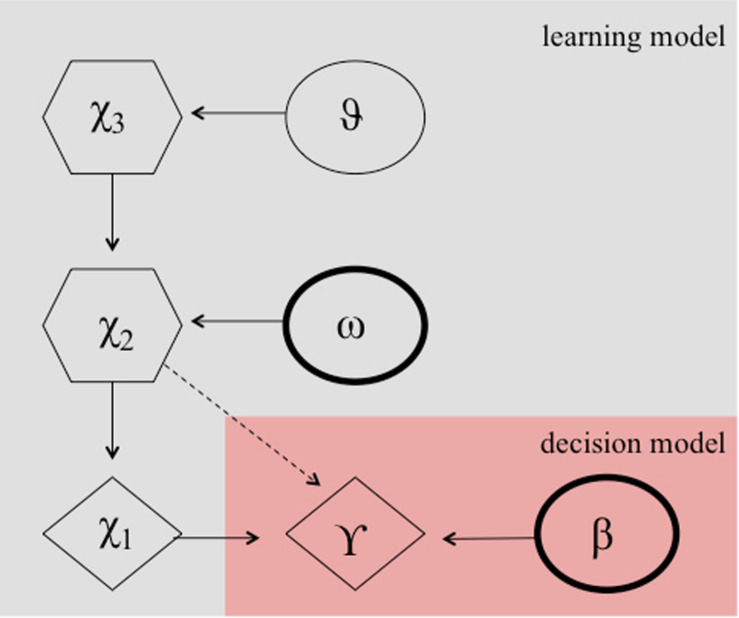
Hierarchical Gaussian filter model according to Hauser et al. (2014). Markovian states are denoted by X^1^ to X^2^ and ω, and β are the free parameters. These parameters determine the actual choice behavior (γ) probablisticly.

It can be argued that the numerous reversals in the paradigm constitute an unstable environment, which requires the learning rate to adapt according to the individuals estimates of the environmental volatility. Perseverative errors, resembling cognitive inflexibility, might then result from either a smaller learning rate, which might be plausible due to a smaller degree of cognitive maturation in children, or a lower capacity to quickly adapt the learning rate to a changing environment or both. Accordingly, a hierarchical Gaussian filter (HGF) model (Mathys et al., 2011, see Figure 3) has been shown to provide the best model fit for explaining behavior during a probabilistic reversal learning task in adolescence (e.g., in comparison to a simple RW model), using an identical task as in the current study (Hauser et al., 2014). Hence, we computed *two* HGF models, one according to Hauser et al. (2014) including the estimation of the model parameter theta ϑ, and one with the model parameter theta ϑ fixed. Our rational for this additional model was that the task structure was not designed with *variance* in volatility, because contingencies always switched after the association was learned. This in turn might preclude an unbiased subjects-specific estimate of theta ϑ. We chose ϑ to be fixed to −3.5066 (empirically derived from the mean estimate of the whole group of participants from the HGF model containing this parameter). In contrast to the more simplistic RW model, the HGF models employ a flexible learning rate, which adapts to changes in the volatility of the environment and according to the beliefs of the participant about the current value of an object. It thus fully complies with the Bayesian brain hypothesis, which assumes that the brain always learns in a Bayes-optimal fashion, given individually different priors (Dayan et al., 1995; Friston, 2010). Note, the exact formulation, the model inversion, and the complete update equations are described elsewhere (Mathys et al., 2011). In short, the HGF model is a generative Bayesian model consisting of a set of probabilistic assumptions governing learning from sensory stimuli. The model describes a hierarchy of three hidden states (*x*_1_,*x*_2_,*x*_3_) that evolve in time as Gaussian random walks. That is, a transition or updating of a state in time is determined probabilistically, with the walk’s step size given by certain parameters and the next highest level’s state within the hierarchy (Mathys et al., 2011). State *x*_*1*_ denotes a binary environmental state, indicating which stimulus is being rewarded. State *x*_*2*_is associated with a kind of internal belief of a value representation (that a target is being rewarded upon choice) and is being transformed to the probability that *x*_*1*_ is rewarded by a logistic sigmoid transformation.

$$p\,\left(x_{1}^{\left(t\right)}|x_{2}^{\left(t\right)}\right)=s\,{\left(x_{2}^{\left(t\right)}\right)}^{x_{1}^{\left(t\right)}}\,{\left(1-s\,\left(x_{2}^{\left(t\right)}\right)\right)}^{1-x_{1}^{\left(t\right)}}$$

with *s*(*x*):=(1/(1+*e*^−*x*^)). State *x*_*2*_ evolves over time and is determined by a Gaussian random walk. The update of state *x*_*2*_ in time from time point t-1 to time point t is characterized by a normal distribution, i.e., at each time point t. The value of $x_{2}^{\left(t\right)}$ is normally distributed with mean $x_{2}^{\left(t-1\right)}$ and variance $e^{k\,x_{3}^{t}+\omega }$

$$p\,\left(x_{2}^{\left(t\right)}\right)∼N\,\left(x_{2}^{\left(t-1\right)},e^{k\,x_{3}^{t}+\omega }\right)$$

Since the variance of this random walk can be taken as a measure of the volatility of *x*_*2*_, the log-volatility $k\,x_{3}^{t}+\omega$ has two components, one phasic and the other tonic: *x*_*3*_ is a state-dependent (phasic) log-volatility, which (together with the free parameter ω, i.e., subject-specific volatility), determines the updating of *x*_*2*_. κ is a scaling factor and was fixed to 1 as in Vossel et al. (2013) and Hauser et al. (2014). The state *x*_*3*_ is normally distributed with mean $x_{3}^{\left(t-1\right)}$ and variance ϑ. ϑ is a free parameter and can be regarded as a subject- specific meta-volatility.

$$p\,\left(x_{3}^{\left(t\right)}\right)∼N\,\left(x_{3}^{\left(t-1\right)},\vartheta \right)$$

The variational inversion of the model yields subject-specific Gaussian belief trajectories about *x*_*2*_ and *x*_*3*_, represented by their means μ_2,_μ_3_and variances (or, equivalently, precisions) σ_2,_σ_3_(π_2,_π_3_). This inversion reveals that the trial-by-trial update equations highly resemble the update equations from RW model:

$$\delta_{1}^{\left(t\right)}=R^{\left(t\right)}-s\,\left({\hat{\mu }}_{2}^{\left(t\right)}\right)$$

where ${\hat{\mu }}_{2}^{\left(t\right)}=\mu_{2}^{\left(t-1\right)}$ is the trial-by-trial mean of the Gaussian prior at the second level and $R^{\left(t\right)}:=x_{1}^{\left(t\right)}.$${\hat{\mu }}_{2}^{\left(t\right)}$ is updated by a precision-weighted RPE

$${\hat{\mu }}_{2}^{\left(t+1\right)}=\mu_{2}^{\left(t\right)}={\hat{\mu }}_{2}^{\left(t\right)}+\sigma_{2}^{\left(t\right)}\,\delta_{1}^{t}$$

where $\sigma_{2}^{\left(t\right)}$ is the trial-by-trial variance at level 2. It can be expressed by a ratio of precision estimates $\hat{\pi }$

$$\sigma_{2}^{\left(t\right)}=\frac{{\hat{\pi }}_{1}^{\left(t\right)}}{{\hat{\pi }}_{2}^{\left(t\right)}\,{\hat{\pi }}_{1}^{\left(t\right)}+1}$$

$${\hat{\pi }}_{2}^{\left(t\right)}:=\frac{1}{\sigma_{2}^{\left(t-1\right)}+e^{\mu_{3}^{\left(t-1\right)}+\omega }}$$

$${\hat{\pi }}_{1}^{\left(t\right)}:=\frac{1}{s\left(\mu_{2}^{\left(t-1\right)}\right)(1-s\left(\mu_{2}^{\left(t-1\right)}\right)}$$

For the update equations at level 3 and for the derivation of the equations, please refer to Mathys et al., 2011.

Both HGF learning models were combined with a softmax decision model [commonly used for reversal learning and decision-making tasks (e.g., Niv et al., 2012; Diaconescu et al., 2014; Hauser et al., 2014, 2017; Boehme et al., 2016)], i.e., a model describing how internal beliefs of value representations are translated into binary decisions (e.g., left or right button press).

$$p\,\left(A\right)=\frac{1}{1+e^{-\beta \,\left(V_{A}-V_{B}\right)}}$$

where *p*(*A*)denotes the probability of choosing object A and β is a free parameter.

Using this combination of learning and decision model, the following free parameters can be estimated: (a) decision parameter β (i.e., the free parameter of the softmax decision function), (b) volatility parameter ω, and (c) meta-volatility parameter ϑ, (fixed to −3.5066 or estimated). (a) The decision function parameter β determines to what extent the internal beliefs of value representations are directly translated into behavior which favors the best option or how strong the choice is influenced by randomness. A smaller β would indicate more “decision noise” or randomness, but implicitly result in stronger behavioral tendencies to explore options which are currently non-favored by the internal model. Thus, β can also be considered an indication of the exploration-exploitation dimension of the participant’s behavior. A higher β would indicate a more frequent exploitation of the best option (Cohen Jonathan and Aston-Jones, 2005; Cohen et al., 2007; Hauser et al., 2014), which we hypothesize might be valid for individuals with less subclinical rigid and repetitive behavior. (b) The subject-specific volatility parameter ω allows for individual differences in the updating of the internal beliefs of stimulus-reward associations and thus governs to what extent new (conflicting) information is used to update the estimation of the prediction strength. A smaller ω would indicate that new information (i.e., received feedback) is used less readily to update existing internal beliefs (i.e., beliefs about which stimulus is being rewarded). (c) The meta-volatility parameter ϑ indicates how variable the state-dependent volatility estimate (*x*_*3*_) is, and thus can be regarded as a subjects’ tendency to believe volatility is changing over time. For both HGF models, all parameters were estimated separately for the three feedback conditions. Furthermore, we also extracted individual “learning rates” (i.e., weighting of RPE updates) per trial and computed the average learning rate per participant. In both HGF models, RPE updating is governed by the trial-by-trial variance at *x*_*2*_ and highly resembles the learning rate α from Rescorla-Wagner models. Taken together, the model parameters of interest and the learning rate indicate how well participants learn and how efficiently they are able to integrate the feedback information to their current beliefs, given a model-based approach.

We performed Bayesian model selection (BMS) for groups across all participants and for each age group separately. To further investigate learning and decision-making impairments, we compared the parameter estimates of the model that performed best across all subjects using Mann-Whitney tests.

#### Behavioral Analyses of Error Patterns

We also performed a standard analysis of error types, to compare against earlier studies (D’Cruz et al., 2013). In addition to the percentage of overall errors, we further distinguished between two specific types of errors: (1) regressive errors are made when participants choose the previously reinforced target (now incorrect choice) after having already chosen the new and correct target at least once, and (2) perseverative errors are made when participants continue to choose the previously reinforced target (now incorrect choice) before they chose the new and correct target. Regressive errors thus indicate failure to retain a newly identified and correct pattern while perseverative errors indicate failure to quickly shift the response after a reversal.

## Results

### Model Comparison

Using Bayesian Model Selection (BMS) for groups (Stephan et al., 2009; Rigoux et al., 2014), we found that the HGF model with meta-volatility parameter ϑ being fixed performed better compared to the HGF including an estimation of the meta-volatility parameter ϑ and the anti-correlated RW model across all subjects as well as for both age groups separately (i.e., children and adolescents; *P*_*x*_ = 0.95; P_*x*_ is the exceedance probability), thus the probability that the HGF including a fixed meta-volatility parameter ϑ performs better than the other two models included in the comparison is 95% (see Table 1).

**TABLE 1:** Exceedence probability for the model comparison between the HGF model with meta-volatility parameter ϑ being fixed, the HGF including an estimation of the meta-volatility parameter ϑ and the anti-correlated RW model.

		**Exceedence Probability**
HGF ϑ being fixed versus RW	Across all conditions	0.95
HGF ϑ being fixed versus HGF ϑ being estimated	Across all conditions	0.99

To further validate our model selection, we aimed to recover the selected model in a simulated data set. We therefore simulated data sets for children and adolescents using the estimated parameters from the wining model and applied the same model fitting and selection procedure as to the actual data set. These additional analyses further confirmed that the HGF including a fixed theta was the best fitting model. For more detailed description and analyses see Supplementary Material.

### Model Parameter Comparison

For a complete overview of the model parameter comparison see Table 2. Mann-Whitney tests comparing the model parameters and average learning rate across participants and conditions revealed that TD children and adolescents showed no significant overall difference in the decision parameter (β: children, 5.96 [4.18]; adolescents, 5.91 [4.01]; *U* = 344; *z* = −1.443; *p* > 0.05).

**TABLE 2:** Model parameter and learning rate comparison using Mann-Whitney tests across participants and conditions.

		**Children**	**Adolescents**	**Significance**
Decision parameter β – mean [SD]	Across all conditions	5.96 [4.18]	5.91 [4.00]	*U* = 344; *z* = −1.443 *p* > 0.05
Subject-specific volatility estimate ω- mean [SD]	Across all conditions	0.77 [0.81]	0.50 [0.79]	*U* = 269; *z* = −1.443 *p* = 0.149
	Control feedback	1.01 [1.13]	0.34 [0.80]	*U* = 222; *z* = −2.281 *p* = 0.023
	Social feedback	0.47 [1.11]	0.58 [0.89]	*U* = 347; *z* = −0.53 *p* > 0.05
	Individualized Feedback	0.83 [1.01]	0.58 [0.87]	*U* = 317; *z* = −0.588 *p* > 0.05
Learning rate - mean [SD]	Across all conditions	0.21 [0.12]	0.26 [0.12]	*U* = 220; *z* = −0.160 *p* > 0.05
	Control feedback	0.18 [0.16]	0.28 [0.12]	*U* = 182; *z* = −2.36 *p* > 0.05
	Social feedback	0.24 [0.12]	0.25 [0.12]	*U* = 291; *z* = −0.417 *p* = 0.018
	Individualized Feedback	0.21 [0.13]	0.26 [0.16]	*U* = 293.5; *z* = −0.585 *p* > 0.05

We also found no significant differences between age groups for the overall subject-specific volatility estimate across conditions (ω: children, 0.77 [0.81]; adolescents, 0.50 [0.79]; *U* = 269; *z* = −1.443; *p* > 0.05). However, detailed inspection of group differences between specific conditions revealed that the subject-specific volatility estimate ω differed between age-groups in the control feedback condition (ω: children, 1.01 [1.13]; adolescents, 0.34 [0.80]; *U* = 222; *z* = −2.281; *p* = 0.023), but less pronounced for the other conditions (individual feedback condition: children 0.83 [1.01] and adolescents 0.58 [0.87]; *U* = 317; *z* = −0.588; *p* > 0.05 and social feedback condition: children 0.47 [1.11] and adolescents 0.58 [0.89]; *U* = 347; *z* = −0.53; *p* > 0.05).

Children and adolescents did not differ in their average learning rate across conditions (children, 0.21 [0.12]; adolescents, 0.26 [0.12]; *U* = 220; *z* = −0.160; *p* > 0.05), but in the control feedback condition (children, 0.18 [0.16]; adolescents, 0.28 [0.12]; *U* = 182; *z* = −2.36; *p* = 0.018), though not in the individual feedback (children, 0.21 [0.13]; adolescents, 0.26 [0.16]; *U* = 293; *z* = −0.585; *p* > 0.05) and social feedback condition (children, 0.24 [0.12]; adolescents, 0.25 [0.12]; *U* = 291; *z* = −0.417; *p* > 0.05).

**FIGURE 4:**
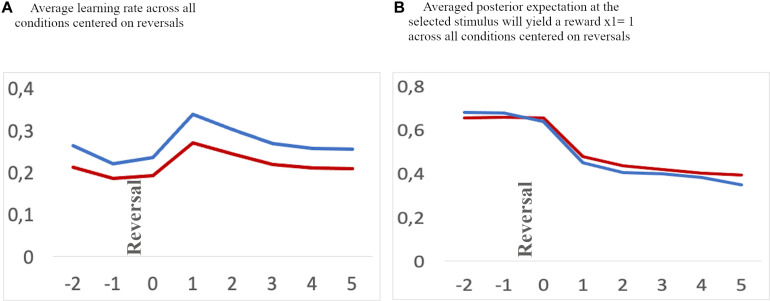
**(A)** Average learning rate. **(B)** Value representation [1: stable internal belief that stimulus **(A)** is being rewarded, and 0: stable internal beliedf that stimulus **(B)** is being rewarded] for a time frame aligned to reversals [two trials before a reversal (–2 and –1), and six trials after a reversal (0–5) across all conditions]. Blue: adolescents, red: children. Note, the values at a specific trial (e.g. 0, the reversal trial) indicate the changes in values after feedback has been received.

**FIGURE 5:**
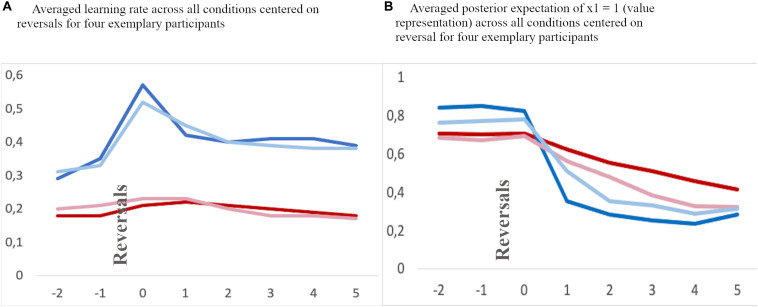
Exemplary averaged learning rate **(A)** and valye representation **(B)** across all feedback conditions for two children (red lines) and two adolescents (blue lines) aligned to reversal trials (“0”) and two trials before and 5 trials thereafter.

To further illustrate the impact of differential model parameters on the dynamics of internal beliefs, we extracted individual estimates of hierarchical states within the HGF model on a trial-by-trial basis. We first extracted the averaged learning rate and the current value representation across conditions aligned to reversal trials and two trials before and six trials thereafter (Figures 4, 5B). We then averaged a sequential series of trials, aligned to reversal trials. The learning rate of adolescents increased rapidly at the time of reversal and decreased quickly thereafter, along with a quick decline in the value representation for the choice of stimulus A. On the other hand, children had a smaller learning rate during reversals, along with a slower value representation update.

### Percentage of Overall Error

**FIGURE 6:**
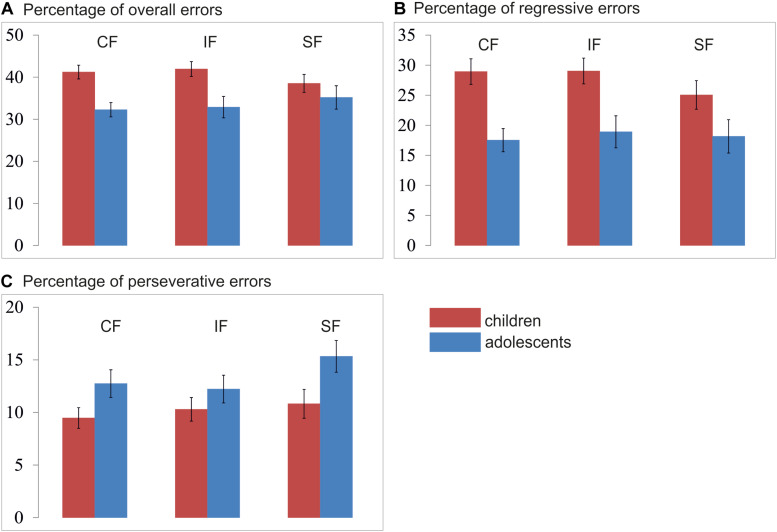
Mean percentage of **(A)** overall errors, **(B)** regressive errors, and **(C)** perseverative errors per condition and age group. CF, control feedback; IF, individualized feedback; SF, social feedback.

All error rates were normally distributed. A mixed 3 × 2 ANOVA analysis with type of feedback (Social/Individualized/Control Feedback) as within-group factor and the between-group factor age-group (Children/Adolescents) revealed a significant feedback x age-group interaction [*F*(2,102) = 5.65; *p* = 0.006] and a main effect for age-group [*F*(1,51) = 8.57; *p* = 0.005], see Figure 6A. *Post hoc* independent-samples *t*-test revealed that children (*M* = 40.42%; SD = 7.95) made significantly more errors than adolescents (*M* = 33.44%; SD = 9.4; *t*(51) = 2.93; *p* = 0.005). Additional *post hoc* independent-samples *t*-tests further revealed that children made significantly more errors in the control (children: *M* = 41.22%; SD = 8.77; adolescents: *M* = 32.27%; SD 7.61; *t*(51) = 3.95; *p* < 0.001) and individual feedback condition (children: *M* = 41.94%; SD = 9.38; adolescents: *M* = 32.87%; SD 11.38; *t*(51) = 3.17; *p* = 0.003), but not in the social feedback condition (children: *M* = 38.12%; SD = 8.51; adolescents: *M* = 33.19%; SD 12.45; *t*(51) = 1.01; *p* = 0.319). There was no order effect, neither for the social [*F*(1,51) = 0.02; *p* = 0.977], or the individual [*F*(1,51) = 0.25; *p* = 0.778] or the control feedback [*F*(1,51) = 0.41; *p* = 0.669].

#### Percentage of Regressive Errors

A regressive error is defined as an error whereby participants choose the previously reinforced response after having already chosen the new and correct choice at least once. The regressive errors thus indicate how well someone retains the new and correct pattern after having identified the new target at least once correctly. A mixed 3 × 2 ANOVA analysis with type of feedback (Social/Individualized/Control Feedback) as a within-group factor and the between-group factor age-group (Children/Adolescents) revealed no significant interaction [*F*(2,102) = 2.27; *p* = 0.114] nor a main effect for condition [*F*(1,102) = 2.74; *p* = 0.074], but a significant main effect for age-group (F1,51) = 10.84; *p* = 0.002) (see Figure 6B). *Post hoc* independent samples *t*-test revealed that children (*M* = 27.67%; SD = 10.99) made significantly more regressive errors than adolescents (*M* = 18.19%; SD = 9.82; *t*(51) = 3.29; *p* = 0.002). There was no order effect, neither for the social [*F*(1,51) = 0.07; *p* = 0.932], or the individual [*F*(1,51) = 1.10; *p* = 0.341] or the control feedback [*F*(1,51) = 0.70; *p* = 0.501].

#### Percentage of Perseverative Errors

A perseverative error is defined as a trial in which participants chose the previously reinforced response despite ongoing negative feedback before they chose the new and correct target. The perseverative errors thus indicate how fast someone shifts the response after a reversal. A mixed 3 × 2 ANOVA analysis with type of feedback (Social/Individualized/Control Feedback) as within-group factors and the between-group factor age-group (Children/Adolescents) revealed no significant interaction [*F*(2,102) = 1.51; *p* = 0.231], but a significant main effect for feedback condition [*F*(2,102) = 3.77; *p* = 0.030] and age-group [*F*(1,51) = 4.67; *p* = 0.035] were observed (see Figure 6C). First, *post hoc* independent-samples *t*-test revealed that adolescents (*M* = 13.43%; SD = 5.30) made significantly more perseverative errors than children across all conditions (*M* = 10.20%; SD = 5.56; *t*(51) = 2.16; *p* = 0.035). Second, *post hoc* paired-sample *t*-test revealed that across age groups significantly more errors were made during the social feedback (*M* = 12.82%; SD = 0.10) than during the control feedback condition (*M* = 10.91%; SD = 0.01; *t*(51) = 2.56; *p* = 0.014) and the individual feedback condition (*M* = 11.11%; SD = 0.06; *t*(51) = 2.14; *p* = 0.037), but no significant difference between the control and individual feedback conditions (*t*(51) = 0.32; *p* = 0.745). There was no order effect, neither for the social [*F*(1,51) = 0.15; *p* = 0.864], or the individual [*F*(1,51) = 1.50; *p* = 0.232] or the control feedback [*F*(1,51) = 1.50; *p* = 0.232].

#### Error Rates in Simulated Models

We further aimed to recover the empirical differences in error rates between children and adolescents in a simulated data set, using the empirically determined mean estimated parameters from the model estimation step (see Supplementary Material for details of the simulation). These recovered error rates revealed a comparable pattern as the empirical error rates described above (see section “Percentage of Regressive Errors” and “Percentage of Perseverative Errors”). That is, adolescents made more perseverative errors than children, and at the same time less regressive errors than children.

### Valence Rating of Feedback Videos

A mixed 3 × 2 × 2 ANOVA analysis with type of feedback (Social/Individualized/Control Feedback) and valence (Rewarding/Non-rewarding) as within-group factors and the between-group factor age-group (Children/Adolescents) revealed a significant main effect for valence [*F*(1,51) = 256.92; *p* < 0.001], but no significant three-way interaction [*F*(1,44) = 0.445; *p* = 0.644], main effect for age-group [*F*(1,51) = 1.84; *p* = 0.182], or main effect of type of feedback [*F*(1,51) = 0.500; *p* = 0.610]. As intended, participants rated the rewarding feedback videos (*M* = 6.99; SD = 1.39) as more rewarding [*t*(51) = 16.05, *p* < 0.001] than the non-rewarding feedback videos (*M* = 2.39; SD = 1.39).

### Correlational Analyses

First, we computed correlational analyses between the (a) extracted model parameters and the learning rate and (b) errors in order to gain further insight into the relation between the model parameters and measurable behavior (i.e., error rates) to ensure face validity of the parameter value interpretations. Second, we computed correlational analyses between extracted model parameters and a clinical measure of “restrictive interests and repetitive behavior” measured by a subscale of the Social Responsiveness parent-questionnaire [SRS-RRB]. This subscale reflects rigid behavior and stereotypes typically observable in children with ASD, but also subclinical variance in typically developing (TD) children.

#### Model Parameters and Errors

The exploration-exploitation estimate β correlated with the percentage total errors (*r* = −0.292; *p* = 0.034), but not with perseverative errors (*r* = 0.114; *p* = 0.421) or regressive errors (*r* = −0.094; *p* = 0.507). The subject-specific volatility estimate ω did not correlate with the percentage of total errors (*r* = −0.11; *p* = 0.424), regressive errors (*r* = −0.23; *p* = 0.109), or perseverative errors (*r* = 0.227; *p* = 105). All results remain significant when Bonferroni corrected. The overall average learning rate correlated negatively with the percentage of total errors (*r* = −0.289; *p* < 0.05), but not with regressive errors (*r* = −0.166; *p* > 0.05) or with perseverative errors (*r* = 0.14; *p* > 0.05).

#### Level of Social Responsiveness (SRS-2)

The SRS Total score was marginally correlated with the parameter β (*r* = 0.27; *p* = 0.059) and the SRS-RRB score was positively correlated with the parameter β (*r* = 0.32; *p* = 0.025), suggesting a link between the shape of the decision function translating value representation into behavior and inflexibility in everyday behavior. The subject-specific volatility estimate ω did not correlate with the SRS-RRB (*r* = 0.01; *p* = 0.954).

## Discussion

Cognitive flexibility is an essential skill that allows us to cope with the demands of a continuously changing environment and moreover is deficient in a broad range of neurodevelopmental and psychiatric disorders. We investigated the (1) developmental differences from childhood to adolescents in probabilistic reversal learning as an index of cognitive flexibility (2) using a computational modeling approach (3) comparing various types of feedback. In addition to mere differences in error rates, the analysis of model parameters provides a more nuanced picture of the psychological processes underlying performance differences during development from childhood to adolescence.

First, children made more overall errors than adolescents, which is in line with previous studies demonstrating an improvement of executive functioning from childhood to adolescence (Blakemore and Choudhury, 2006), including decision-making (Crone et al., 2003; Kerr and Zelazo, 2004; Overman, 2004; Van Duijvenvoorde et al., 2012), cognitive flexibility (Crone et al., 2004; Alvarez and Emory, 2006; Zelazo and Carlson, 2012), and (probabilistic) feedback learning (Van Duijvenvoorde et al., 2008; Eppinger and Kray, 2009; Hämmerer and Eppinger, 2012; Van Duijvenvoorde et al., 2013). However, the exact mechanisms underlying developmental differences in cognitive flexibility from childhood to adolescence to date remain poorly understood and are difficult to infer from error patterns alone. Hence, previous studies provided various explanations for behavioral differences in cognitive flexibility during development, such as difficulties in distinguishing informative and non-informative feedback (see for a discussion: Kirkham and Diamond, 2003), differences in monitoring (Davies et al., 2004; van Leijenhorst et al., 2006), inhibitory (Huizinga et al., 2006) or cognitive control mechanisms (Van Duijvenvoorde et al., 2008). Modeling approaches might thus help to understand how participants integrate information throughout the task by providing a more stringent mapping between model parameters and assumed psychological processes. Specifically, the HGF modeling has been successfully used to infer mechanisms underlying cognitive flexibility (Jiang et al., 2014) under multiple forms of uncertainty (e.g., perceptual uncertainty and environmental volatility) (Mathys et al., 2011). The HGF approach can model how the brain flexibly integrates information across different time scales to predict change by dynamically updating predictions based on integrating past information with recent observations (see for review: Jiang et al., 2014). In the specific case of probabilistic reversal learning, this implies that the participants use the given feedback to improve future choices via maximization of favorable outcomes.

Second and with respect to the parameter ω, our results suggest that children had a bias toward a slower update of their estimation of the prediction strength for a rewarding outcome than adolescents as reflected by a smaller individual volatility parameter in the control feedback condition. This is also related to a smaller learning rate in the control feedback condition (see section “Model Parameter Comparison” and Figure 4A for the learning rate specifically around a reversal). Thus, our data suggest that children use new and conflicting information less readily and less immediately than adolescents to update their internal beliefs of stimulus-reward associations, resulting in less efficient learning in the context of an unstable environment (see Figure 4), specifically when the feedback is a simple cross or check mark (control feedback). This conclusion is consistent with previous accounts suggesting a less efficient updating based on feedback (Eppinger and Kray, 2009; Hämmerer and Eppinger, 2012; Van Duijvenvoorde et al., 2013, 2008). It is plausible that this mechanism also underlies the higher overall and the higher regressive error rate in children as compared to adolescents. Children, who update their internal beliefs of stimulus-reward association slower, are also likely to make more errors. Note that on the other hand a “too fast” updating could also result in increased number of errors. Thus, it is rather necessary to have an optimal updating “speed.” This is particularly relevant for regressive errors in later stages after a reversal, since with slower learning rates children are slower in reaching a level of internal beliefs where the correct option is clearly represented as favorable (see Figures 4A,B). In other words, they seem to have a less stable representation of the stimulus-reward association, which in turn might result in more changes of response choice and hence in less preservative but more regressive errors. The findings of (i) the negative correlation between overall average learning rate and the number of total and regressive errors, but (ii) no correlation with perseverative errors as well as (iii) the negative correlation between the parameter ω and the number of total and regressive errors, but (iv) positive correlation with perseverative errors further substantiates this conclusion. On the other hand, fast updating in adolescents after each reversal results in relatively few overall errors as well as few regressive errors since the correct option is clearly represented as favorable soon after a reversal with only a short period of “doubt” (see Figures 4, 5).

Third, slight differences between conditions could be observed. First, the difference between children and adolescents was most pronounced for both total number of errors and the parameter ω during the control feedback condition. Second, with respect to overall errors we found less prominent differences between children and adolescents during the social feedback conditions. Third, we also found more perseverative errors during social feedback compared to control and individualized feedback across age-groups. On the one hand, it might be hypothesized that specifically in real life settings social feedback is typically more consistent and that contingencies usually do not change as fast as during this laboratory setting, resulting in slower updating of a value contingency. On the other hand, it might also be plausible that social feedback generates a stronger association resulting in more “difficulties” to reverse an association and learn a new one. Presumably, additional motivational mechanisms may come into play for social and individual feedback conditions whereas the control feedback condition might best reflect “pure” reversal learning to reveal general mechanisms.

Lastly, we observed a relationship between external measures of everyday behavior and modeling parameters, suggesting that our findings may extend, at least to a certain degree, beyond the specific paradigm in a laboratory setting. We found a correlation between the specific decision-making model parameter beta and a measure of rigidity in everyday life (i.e., SRS-RBB). Thus, there could be a link between rigidity and a tendency to always stick to the potentially best option as favored by an acquired internal model, i.e., exploit the inferred contingencies. This is in line with the interpretation of the parameter beta in terms of implicit exploration/exploitation behavior (Cohen Jonathan and Aston-Jones, 2005; Cohen et al., 2007; Hauser et al., 2014) and would be a maladaptive behavior in highly volatile environments. This relationship may provide one possible explanation of how impairments in cognitive flexibility translate into behavior. Further studies in patients with high levels of behavioral rigidity (e.g., Obsessive-compulsive disorder, Autism spectrum disorder or Anorexia nervosa) are needed to explore this hypothesis.

Taken together, we could show for the first time that hierarchical Bayesian modeling (here: HGF-model) is a valid approach to assess developmental effects in reversal learning. We observed that the subject-specific volatility parameter changes during typical development from childhood to adolescence and that children in general have a smaller learning rate. Differences in these parameters may explain the typical differences in error patterns in children and adolescents during a probabilistic reversal learning task, and are associated with overall cognitive flexibility. That is, children might be less sure which stimulus will most probably be rewarded, because they use the feedback less readily and immediately to adapt their behavior. Adolescents, on the other hand, used the feedback they received in a more efficient way, since after having established stronger stimulus response associations they changed their response choice when their stimulus-reward association had been updated quickly and efficiently. In line with this interpretation, Van Duijvenvoorde et al. (2013) showed that children and adolescents are not generally impaired in probabilistic feedback monitoring, but are specifically impaired in updating their stimulus-reward association based on received probabilistic feedback.

Developmental differences in performance on decision-making or feedback learning have often been attributed to less mature frontal brain development in children, resulting in less developed control, monitoring and/or inhibitory capabilities in children compared to adolescents (e.g., Crone et al., 2004; Davies et al., 2004; van Leijenhorst et al., 2006), suggesting a top-down modulation of learning. Our results support the idea of developmental differences in hierarchical top-down processing, since we observed superiority of the HGF model in comparison to a simple bottom-up learning model in both age-groups, but further development and “shaping” of the model parameters with increasing age. The latter may be associated with cortical maturation or accumulated experience (e.g., estimating the environment as more volatile in adolescence than in childhood) that occurs from childhood to adolescence.

Yet, in future investigations, neuroimaging methods such as functional magnetic resonance imaging (fMRI) should be used to reveal differential developmental neural mechanisms associated with respective model parameters. Previous studies have attributed differences in probabilistic feedback learning to regional and connectivity changes of the prefrontal cortex (Hämmerer and Eppinger, 2012; van den Bos et al., 2012) that undergoes prominent changes during adolescence (e.g., Paus, 2005; Crone and Dahl, 2012; Konrad et al., 2013) and is associated with cognitive control, monitoring, and inhibitory mechanisms. However, it would be crucial to investigate to what extent the Bayesian modeling approach may add to that knowledge by relating developmental changes in model parameters to specific functional brain changes. For example, Hauser et al. (2015b) demonstrated that adolescents and adults performed comparably in a probabilistic reversal learning task. However, differences in modeling parameters indicated that adolescents learned faster from negative feedback, which was associated with altered brain activation within the anterior insula.

Furthermore, the modeling approach to reversal learning is particularly promising to study participants with various neurodevelopmental and/or psychiatric disorders to investigate atypical development and the disturbance of cognitive flexibility and reward processing during development and its impact on various domains. Specifically, we found a correlation between the model parameter beta and a measure of subclinical rigidity in everyday life. Children and adolescents with varying psychiatric disorders, including ASD, OCD, and ADHD, show deficits in cognitive flexibility and reward processing (Demurie et al., 2011; Kohls et al., 2011; Hauser et al., 2014; Verfaillie et al., 2016; Tezcan et al., 2017). Appropriate model-based approaches provide the opportunity to augment standard behavioral analyses and provide a closer link to underlying psychological mechanisms and their underlying neural substrates.

## Data Availability Statement

The datasets generated for this study are available on request to the corresponding author.

## Ethics Statement

The studies involving human participants were reviewed and approved by the Ethics Committee of the University Hospital RWTH Aachen. Written informed consent to participate in this study was provided by the participants’ legal guardian.

## Author Contributions

EW: project planning, data acquisition and analysis, and manuscript completion. JK: project planning, data acquisition, and significant contribution to the manuscript completion. GF: significant contribution to the project planning and manuscript completion. BH-D: significant contribution to participant recruitment and manuscript completion. KK: significant contribution to project planning, participant recruitment, and manuscript completion. MS-R: project planning, data analysis, manuscript completion, and supervision of the study. All authors contributed to the article and approved the submitted version.

## Conflict of Interest

The authors declare that the research was conducted in the absence of any commercial or financial relationships that could be construed as a potential conflict of interest.

## Footnotes

1 http://www.neurobs.Com

2 https://osf.io/6hbw4

## References

[B1] Achenbach T. M. (1991). *Manual for the Child Behavior Checklist/4-18 and 1991 profile.* Burlington, VT: Department of Psychiatry, University of Vermont.

[B2] Alvarez J. A. Emory E. (2006). Executive function and the frontal lobes: a meta-analytic review. *Neuropsychol. Rev.* 16 17–42. 10.1007/s11065-006-9002-x 16794878

[B3] Blakemore S.-J. Choudhury S. (2006). Development of the adolescent brain: implications for executive function and social cognition. *J. Child Psychol. Psychiatry Allied Discip.* 47 296–312. 10.1111/j.1469-7610.2006.01611.x 16492261

[B4] Blakemore S.-J. Robbins T. W. (2012). Decision-making in the adolescent brain. *Nat. Neurosci.* 15 1184–1191. 10.1038/nn.3177 22929913

[B5] Boehme R. Lorenz R. C. Gleich T. Romund L. Pelz P. Golde S. (2016). Reversal learning strategy in adolescence is associated with prefrontal cortex activation. *Eur. J. Neurosci.* 45 129–137. 10.1111/ejn.13401 27628616

[B6] Buelow M. T. Amick M. M. Queller S. Stout J. C. Friedman J. H. Grace J. (2015). Feasibility of use of probabilistic reversal learning and serial reaction time tasks in clinical trials of Parkinson’s disease. *Parkinsonism Related Disord.* 21 894–898. 10.1016/j.parkreldis.2015.05.019 26040709

[B7] Chevallier C. Tonge N. Safra L. Kahn D. Kohls G. Miller J. (2016). Measuring social motivation using signal detection and reward responsiveness. *PLoS One* 11:167024. 10.1371/journal.pone.0167024 27907025 PMC5132309

[B8] Cohen J. D McClure S. M. Yu A. J. (2007). Should I stay or should I go? how the human brain manages the trade-off between exploitation and exploration. *Philosophical Transac. Royal Soc. B: Biol. Sci.* 362 933–942. 10.1098/rstb.2007.2098 17395573 PMC2430007

[B9] Cohen Jonathan D. Aston-Jones G. (2005). Cognitive neuroscience: decision amid uncertainty. *Nature* 436 471–472. 10.1002/adma.200401726, 16049461

[B10] Constantino J. N. Davis S. A Todd R. D. Schindler M. K. Gross M. M. Brophy S. L. (2003). Validation of a brief quantitative measure of autistic traits: comparison of the social responsiveness scale with the autism diagnostic interview-revised. *J. Autism Dev. Disord.* 33 427–433. 12959421 10.1023/a:1025014929212

[B11] Cools R. Clark L. Owen A. M. Robbins T. W. (2002). Defining the neural mechanisms of probabilistic reversal learning using event-related functional magnetic resonance imaging. *J. Neurosci.* 22 4563–4567. 10.1523/jneurosci.22-11-04563.2002 12040063 PMC6758810

[B12] Costescu C. A. Vanderborght B. David D. O. (2014). Reversal learning task in children with autism spectrum disorder: a robot-based approach. *J. Autism Dev. Disord.* 45 3715–3725. 10.1007/s10803-014-2319-z 25479815

[B13] Crawley D. Zhang L. Jones E. J. H. Ahmad J. José Cáceres A. S. Oakley B. (2019). Modeling cognitive flexibility in autism spectrum disorder and typical development reveals comparable developmental shifts in learning mechanisms. *PsyArXiv* 2019 1–34. 10.31234/osf.io/h7jcm

[B14] Crone E. A. Dahl R. E. (2012). Understanding adolescence as a period of social – affective engagement and goal flexibility. *Nature* 13 636–650. 10.1038/nrn3313 22903221

[B15] Crone E. A. Ridderinkhof K. R. Worm M. Somsen R. J. M. van der Molen M. W. (2004). Switching between spatial stimulus response mappings: a developmental study of cognitive flexibility. *Dev. Sci.* 7 443–455. 10.1111/j.1467-7687.2004.00365.x 15484593

[B16] Crone E. A. van der Molen M. W. (2004). Developmental changes in real life decision making: performance on a gambling task previously shown to depend on the ventromedial prefrontal cortex. *Dev. Neuropsychol.* 25 251–279. 10.1207/s15326942dn2503 15147999

[B17] Crone E. A. Van Der Veen F. M. Van Der Molen M. W. Somsen R. J. M. Van Beek B. Jennings J. R. (2003). Cardiac concomitants of feedback processing. *Biol. Psychol.* 64 143–156. 10.1016/S0301-0511(03)00106-6 14602359

[B18] Culbreth A. J. Gold J. M. Cools R. Barch D. M. (2016). Impaired activation in cognitive control regions predicts reversal learning in schizophrenia. *Schizophrenia Bull.* 42 484–493. 10.1093/schbul/sbv075 26049083 PMC4753588

[B19] Davies P. L. Segalowitz S. J. Gavin W. J. (2004). Development of response-monitoring ERPs in 7- to 25-Year-olds patricia. *Dev. Neuropsychol.* 25 355–376. 10.1207/s15326942dn2503_6 15148003

[B20] Dayan P. Hinton G. E. E. Neal R. M. M. Zemel R. S. S. (1995). The helmholtz machine. *Neural. Comp.* 7 889–904. 10.1162/neco.1995.7.5.889 7584891

[B21] D’Cruz A. Ragozzino M. E. Mosconi M. W. Shrestha S. Cook E. H. Sweeney J. A. (2013). Reduced behavioral flexibility in autism spectrum disorder. *Neuropsychology* 27 152–160. 10.1016/j.biotechadv.2011.08.021.Secreted 23527643 PMC3740947

[B22] D’Cruz A.-M. Mosconi M. W. Ragozzino M. E. Cook E. H. Sweeney J. A. (2016). Alterations in the functional neural circuitry supporting flexible choice behavior in autism spectrum disorders. *Translational. Psychiatry* 6:e916. 10.1038/tp.2016.161 27727243 PMC5315543

[B23] Demurie E. Roeyers H. Baeyens D. Sonuga-Barke E. (2011). Common alterations in sensitivity to type but not amount of reward in ADHD and autism spectrum disorders. *J. Child Psychol. Psychiatry Allied Discip.* 52 1164–1173. 10.1111/j.1469-7610.2010.02374.x 21223259

[B24] Diaconescu A. O. Mathys C. Weber L. A. E. Daunizeau J. Kasper L. Lomakina E. I. (2014). Inferring on the intentions of others by hierarchical bayesian learning. *PLoS Comp. Biol.* 10:e1003810. 10.1371/journal.pcbi.1003810 25187943 PMC4154656

[B25] Eppinger B. Kray J. (2009). To choose or to avoid: age differences in learning from positive and negative feedback. *J. Cogn. Neurosci.* 23 41–52. 10.1162/jocn.2009.21364 19925176

[B26] Friston K. (2010). The free-energy principle: a unified brain theory? *Nat.Rev. Neurosci.* 11 127–138. 10.1038/nrn2787 20068583

[B27] Ghahremani D. G. Monterosso J. Jentsch J. D. Bilder R. M. Poldrack R. A. (2010). Neural components underlying behavioral flexibility in human reversal learning. *Cerebral Cortex*, 20 1843–1852. 10.1093/cercor/bhp247 19915091 PMC2901019

[B28] Gläscher J. Hampton A. N. O’Doherty J. P. (2009). Determining a role for ventromedial prefrontal cortex in encoding action-based value signals during reward-related decision making. *Cerebral Cortex*, 19 483–495. 10.1093/cercor/bhn098 18550593 PMC2626172

[B29] Hämmerer D. Eppinger B. (2012). Dopaminergic and prefrontal contributions to reward-based learning and outcome monitoring during child development and aging. *Dev. Psychol.* 48 862–874. 10.1037/a0027342 22390655

[B30] Hauser T. U. Iannaccone R. Ball J. Mathys C. Brandeis D. Walitza S. (2014). Role of the medial prefrontal cortex in impaired decision making in juvenile attention-deficit/hyperactivity disorder. *JAMA Psychiatry* 71 1165–1173. 10.1001/jamapsychiatry.2014.1093 25142296

[B31] Hauser T. U. Hunt L. T. Iannaccone R. Walitza S. Brandeis D. Brem S. (2015a). Temporally dissociable contributions of human medial prefrontal subregions to reward-guided learning. *J. Neurosci.* 35 11209–11220. 10.1523/JNEUROSCI.0560-15.2015 26269631 PMC4532755

[B32] Hauser T. U. Iannaccone R. Walitza S. Brandeis D. Brem S. (2015b). Cognitive flexibility in adolescence: neural and behavioral mechanisms of reward prediction error processing in adaptive decision making during development. *NeuroImage* 104 347–354. 10.1016/j.neuroimage.2014.09.018 25234119 PMC4330550

[B33] Hauser T U Iannaccone R. Dolan R. J. Ball J. Hättenschwiler J. Drechsler R. (2017). Increased fronto-striatal reward prediction errors moderate decision making in obsessive – compulsive disorder. *Psychol. Med.* 47 1246–1258. 10.1017/S0033291716003305 28065182

[B34] Huizinga M. Dolan C. V. van der Molen M. W. (2006). Age-related change in executive function: developmental trends and a latent variable analysis. *Neuropsychologia* 44 2017–2036. 10.1016/j.neuropsychologia.2006.01.010 16527316

[B35] Ionescu T. (2012). Exploring the nature of cognitive flexibility. *N. Ideas Psychol.* 30 190–200. 10.1016/j.newideapsych.2011.11.001

[B36] Izquierdo A. Brigman J. L. Radke A. K. Rudebeck P. H. Holmes A. (2016). The neural basis of reversal learning: an updated perspective. *Neuroscience* 345 12–26. 10.1016/j.neuroscience.2016.03.021 26979052 PMC5018909

[B37] Jiang J. Heller K. Enger T. (2014). Bayesian modeling of flexible cognitive control. *Neurosci. Biobehav. Rev.* 46 30–43. 10.1007/s12020-009-9266-z.A 24929218 PMC4253563

[B38] Kerr A. Zelazo P. D. (2004). Development of “hot” executive function: the children’s gambling task. *Brain Cogn.* 55 148–157. 10.1016/S0278-2626(03)00275-6 15134849

[B39] Kirkham N. Z. Diamond A. (2003). Sorting between theories of perseveration: performance in conflict tasks requires memory, attention and inhibition. *Dev. Sci.* 6 474–476. 10.1111/1467-7687.00303

[B40] Kohls G. Peltzer J. Herpertz-Dahlmann B. Konrad K. (2009). Differential effects of social and non-social reward on response inhibition in children and adolescents. *Dev. Sci.* 12 614–625. 10.1111/j.1467-7687.2009.00816.x 19635087

[B41] Kohls G. Peltzer J. Schulte-Rüther M. Kamp-Becker I. Remschmidt H. Herpertz-Dahlmann B. (2011). Atypical brain responses to reward cues in autism as revealed by event-related potentials. *J. Autism Dev. Disord.* 41 1523–1533. 10.1007/s10803-011-1177-1 21290174

[B42] Kohls G. Perino M. T. Taylor J. M. Madva E. N. Cayless S. J. Troiani V. (2013). The nucleus accumbens is involved in both the pursuit of social reward and the avoidance of social punishment. *Neuropsychologia* 51 2062–2069. 10.1016/j.neuropsychologia.2013.07.020 23911778 PMC3799969

[B43] Konrad K. Firk C. Uhlhaas P. J. (2013). Hirnentwicklung in der adoleszenz. *Deutsches Arzteblatt Inter.* 110 425–431. 10.3238/arztebl.2013.0425 23840287 PMC3705203

[B44] Landry O. Al-Taie S. (2016). A meta-analysis of the wisconsin card sort task in autism. *J. Autism Dev. Disord.* 46 1220–1235. 10.1007/s10803-015-2659-3 26614085

[B45] Lionello-DeNolf K. M. McIlvane W. J. Canovas D. S. Souza de D. G. Barros R. S. (2010). Reversal learning set and functional equivalence in children with and without autism. *Psychol. Rec.* 58 15–36. 10.1111/j.1600-6143.2008.02497.x.Plasma PMC2828151 20186287

[B46] Luking K. R. Luby J. L. Barch D. M. (2014). Developmental cognitive neuroscience kids, candy, brain and behavior: age differences in responses to candy gains and losses. *Accident Analysis Prev.* 9 82–92. 10.1016/j.dcn.2014.01.005 24534632 PMC4061265

[B47] Mathys C. Daunizeau J. Friston K. J. Stephan K. E. (2011). A bayesian foundation for individual learning under uncertainty. *Front. Hum. Neurosci.* 5:39. 10.3389/fnhum.2011.00039 21629826 PMC3096853

[B48] Nashiro K. Sakaki M. Nga L. Mather M. (2012). Differential brain activity during emotional versus nonemotional reversal learning. *J. Cogn. Neurosci.* 24 1794–1805. 10.1162/jocn_a_00245 22621263 PMC3588885

[B49] Nickchen K. Boehme R. del Mar Amador M. Hälbig T. D. Dehnicke K. Panneck P. (2016). Reversal learning reveals cognitive deficits and altered prediction error encoding in the ventral striatum in Huntington’s disease. *Brain Imaging Behav.* 11 1862–1872. 10.1007/s11682-016-9660-0 27917451

[B50] Niv Y. Edlund J. A. Dayan P. O’Doherty J. P. (2012). Neural prediction errors reveal a risk-sensitive reinforcement-learning process in the human brain. *J. Neurosci.* 32 551–562. 10.1523/JNEUROSCI.5498-10.2012 22238090 PMC6621075

[B51] Overman W. H. (2004). Sex differences in early childhood, *adolescence*, and adulthood on cognitive tasks that rely on orbital prefrontal cortex. *Brain Cogn.* 55 134–147. 10.1016/S0278-2626(03)00279-3 15134848

[B52] Palminteri S. Kilford E. J. Coricelli G. Blakemore S. (2016). The computational development of reinforcement learning during adolescence. *PLoS Comput. Biol.* 12:e1004953. 10.1371/journal.pcbi.1004953 27322574 PMC4920542

[B53] Paus T. (2005). Mapping brain maturation and cognitive development during adolescence. *Trends Cogn. Sci.* 9 60–68. 10.1016/j.tics.2004.12.008 15668098

[B54] Reddy L. F. Waltz J. A. Green M. F. Wynn J. K. Horan W. P. (2016). Probabilistic reversal learning in schizophrenia: stability of deficits and potential causal mechanisms. *Schizophrenia Bull.* 42 942–951. 10.1093/schbul/sbv226 26884546 PMC4903059

[B55] Remijnse P. L. Nielen M. M. A Uylings H. B. M. Veltman D. J. (2005). Neural correlates of a reversal learning task with an affectively neutral baseline: An event-related fMRI study. *NeuroImage* 26(2), 609–618. 10.1016/j.neuroimage.2005.02.009 15907318

[B56] Rescorla R. A. Wagner A. R. (1972). A Theory of Pavlovian Conditioning: Variations in the Effectiveness of Reinforcement and Nonreinforcement. In Black A. H. Prokasy W. F. (Eds.), *Classical conditioning II: current research and theory* (pp. 64–99). New York: Appleton-Century-Crofts.

[B57] Rigoux L. Stephan K. E. Friston K. J. Daunizeau J. (2014). Bayesian model selection for group studies - Revisited. *NeuroImage* 84 971–985. 10.1016/j.neuroimage.2013.08.065 24018303

[B58] Schuch S. Konrad K. (2017). Investigating task inhibition in children versus adults: a diffusion model analysis. *J. Exp. Child Psychol.* 156 143–167. 10.1016/j.jecp.2016.11.012 28068551

[B59] Somerville L. H. Jones R. M. Casey B. J. (2010). A time of change: behavioral and neural correlates of adolescent sensitivity to appetitive and aversive environmental cues. *Brain Cogn.* 72 124–133. 10.1016/j.bandc.2009.07.003 19695759 PMC2814936

[B60] South M. Ozonoff S. Mcmahon W. M. (2005). Repetitive behavior profiles in asperger syndrome and high-functioning autism. *J. Autism Dev. Disord.* 35 145–158. 10.1007/s10803-004-1992-8 15909401

[B61] Stavropoulos K. K. M. Carver L. J. (2014). Reward anticipation and processing of social versus nonsocial stimuli in children with and without autism spectrum disorders, 12 1398–1408. 10.1111/jcpp.12270 24890037

[B62] Stephan K. E. Penny W. D. Daunizeau J. Moran R. J. Friston K. J. (2009). Bayesian model selection for group studies. *NeuroImage* 46 1004–1017. 10.1016/j.neuroimage.2009.03.025 19306932 PMC2703732

[B63] Tezcan D. Tumkaya S. Bora E. (2017). Reversal learning in patients withobsessivecompulsivedisorder (Ocd) and their unaffected relatives: is orbitofrontal dysfunction an endophenotype of ocd? *Psychiatry Res.* 252 231–233. 10.1016/j.psychres.2017.03.001 28285250

[B64] van den Bos W. Cohen M. X. Kahnt T. Crone E. A. (2012). Striatum-medial prefrontal cortex connectivity predicts developmental changes in reinforcement learning. *Cerebral Cortex* 22 1247–1255. 10.1093/cercor/bhr198 21817091 PMC6283353

[B65] Van Der Schaaf M. E. Warmerdam E. Crone E. A. Cools R. (2011). Distinct linear and non-linear trajectories of reward and punishment reversal learning during development: relevance for dopamine’s role in adolescent decision making. *Dev. Cogn. Neurosci.* 1 578–590. 10.1016/j.dcn.2011.06.007 22436570 PMC6987536

[B66] Van Duijvenvoorde A. C. K. Jansen B. R. J. Bredman J. C. Huizenga H. M. (2012). Age-related changes in decision making: comparing informed and noninformed situations. *Dev. Psychol.* 48 192–203. 10.1037/a0025601 21967563

[B67] Van Duijvenvoorde A. C. K. Jansen B. R. J. Griffioen E. S. Van der Molen M. W. Huizenga H. M. (2013). Decomposing developmental differences in probabilistic feedback learning: a combined performance and heart-rate analysis. *Biol. Psychol.* 93 175–183. 10.1016/j.biopsycho.2013.01.006 23352569

[B68] Van Duijvenvoorde A. C. K. Zanolie K. Rombouts S. A. R. B. Raijmakers M. E. J. Crone E. A. (2008). Evaluating the negative or valuing the positive? neural mechanisms supporting feedback-based learning across development. *J. Neurosci.* 28(38), 9495–9503. 10.1523/JNEUROSCI.1485-08.2008 18799681 PMC6671119

[B69] van Leijenhorst L. Crone E. A. Bunge S. A. (2006). Neural correlates of developmental differences in risk estimation and feedback processing. *Neuropsychologia* 44 2158–2170. 10.1016/j.neuropsychologia.2006.02.002 16574168

[B70] Verfaillie S. C. J. de Wit S. J. Vriend C. Remijnse P. L. Veltman D. J. van den Heuvel O. A. (2016). The course of the neural correlates of reversal learning in obsessive-compulsive disorder and major depression: a naturalistic follow-up fMRI study. *J. Obsessive-Compulsive Related Disord.* 9 51–58. 10.1016/j.jocrd.2016.02.004

[B71] Vossel S. Mathys C. Markus Bauer J. D. Driver J. Friston K. J. Stephan K. E. (2013). Spatial attention, precision, and bayesian inference: a study of saccadic response speed. *Cerebral Cortex* 24 1436–1450. 10.1093/cercor/bhs418 23322402 PMC4014178

[B72] Weeda W. D. Van der Molen M. W. Barceló F. Huizinga M. (2014). A diffusion model analysis of developmental changes in children’s task switching. *J. Exp. Child Psychol.* 126 178–197. 10.1016/j.jecp.2014.05.001 24945684

[B73] Weiß R. H. (2006). *Grundintelligenztest Skala 2 - Revision (CFT 20-R): mit Wortschatztest und Zahlenfolgentest - Revision (WS/ZF-R).* Göttingen: Hogrefe.

[B74] Yurgelun-Todd D. (2007). Emotional and cognitive changes during adolescence. *Curr. Opinion Neurobiol.* 17 251–257. 10.1016/j.conb.2007.03.009 17383865

[B75] Zelazo P. D. Carlson S. M. (2012). Hot and cool executive function in childhood and adolescence: development and plasticity. *Child Dev. Perspec.* 6 354–360. 10.1111/j.1750-8606.2012.00246.x

