# Supplementary material for: Developmental Differences in Probabilistic Reversal Learning: A Computational Modeling Approach
Source: Front Neurosci. 2021 Jan 18;14:536596. doi: 10.3389/fnins.2020.536596 (PMC7848134; doi:10.3389/fnins.2020.536596)
Supplement: Supplementary file 1 [file Data_Sheet_1.DOCX]

Supplementary Material

Title: Developmental trajectories of probabilistic reversal learning: a computational modeling approach

Oberwelland Weiss, E.^126^, Kruppa, J.A.^126^, Fink, G.R.^23^, Herpertz-Dahlmann, B.^4^, Konrad, K.^256^, Schulte-Rüther, M.^126^

1. Simulation Analyses

To demonstrate the validity of the model selection procedure as well as unbiased parameter estimation within the winning model (i.e. test for ‘parameter recovery’), we first used Bayesian Model Selection (BMS) for groups (Rigoux, Stephan, Friston, & Daunizeau, 2014; Stephan, Penny, Daunizeau, Moran, & Friston, 2009), as described for the original data in the manuscript (see section 3.1. Model Comparison), and then attempted to recover the empirically determined differences in parameters between children and adolescents in simulated data sets and to validate the model selection. We performed respective ex-post simulations of 20 new population data sets using 1) the parameters (ω and β) of our winning model (i.e., HGF with a fixed theta) as estimated from the actual data for each group (i.e. for children ω=-3.49 and β= 5.28 and for adolescents, ω=-2.30 and β= 5.59) and 2) and applied the same model fitting and selection procedure as to our actual data set. For the simulations, the exact same stimulus and contingency mappings of our original participants were used, but the choice of button press was simulated. Each new population dataset contained 28 simulated adolescent and 25 child participants, as in our actual dataset.

1. 1 Model selection

Using BMS for groups, we confirmed with our simulated data sets that the HGF (theta fixed) performed better compared than the RW model across all subjects (P_x_ = 0.98; P_x_ is the exceedance probability). We used additional cross-validation of model selection using both RW and HGF (theta fixed) simulated data sets with both RW and HGF (theta fixed) fitting procedures, respectively.

Using simulated datasets based on the HGF (theta fixed) model we could confirm that a new model selection again clearly favored HGF (theta fixed) over the RW model (P_x_ = 0.97). Using simulated data sets based on the RW model, model selection revealed a slight advantage for RW over HGF (theta fixed) (P_x_ = 0.56). Overall, these results confirm adequate model recovery from simulated population data sets, lending credence to our choice of BMS procedure.

1. 2 Parameter extraction and calculation simulated data sets

For parameter recovery, we refitted parameters for each of the simulated datasets using the winning model (HGF with a fixed theta, see section 2.3.1 Computational Models). We then extracted the same model parameters (ω and β) and the learning rate and analyzed between-group differences as described in the manuscript (see section 3.2 Model Parameter Comparison) for each of the 20 data sets. In all simulated data sets, between-group differences between children and adolescents were found for both model parameters (ω and β) as well as the learning rate across feedback condition.

For an unbiased estimate of the true population differences, we averaged the parameter and learning rate means across simulated population data sets and compared the between-group differences for all variables. As expected, the simulated population means between children and adolescents differed significantly for both model parameters and the learning rate and were very similar to those parameters extracted from the single empirical data set. Furthermore, population mean estimates for children had a significantly smaller decision parameter β (β: children, 8.19 [1.44]; adolescents, 20.34 [2.12]; p = 0.00) and learning rate (children, 0.09 [0.001]; adolescents, 0.18 [0.004]; p = .00), but a greater subject-specific volatility estimate (ω: children, −4.75 [0.07]; adolescents, −2.83 [0.065]; p = 0.00).
